# Evaluating Biocompounds in Discarded Beetroot (*Beta vulgaris*) Leaves and Stems for Sustainable Food Processing Solutions

**DOI:** 10.3390/foods13162603

**Published:** 2024-08-20

**Authors:** Carolina Mella, Natalia Rojas, Hector Calderon-Bravo, Loreto A. Muñoz

**Affiliations:** 1 Nutrition and Dietetics, Faculty of Medicine and Health Science, Universidad Central de Chile, Coquimbo 1710164, Chile; carolina.mella@ucentral.cl (C.M.); natalia.rojas@ucentral.cl (N.R.); 2 Food Science Lab, Faculty of Medicine and Health Science, Universidad Central de Chile, Santiago 8330546, Chile; hector.calbravo@gmail.com

**Keywords:** beetroot, *Beta vulgaris* L., bioactive compounds, beet leaves and stems

## Abstract

The current trend focuses on reducing food waste, with scientific studies exploring the nutritional value of discarded food components to identify potential health benefits. Beetroot (*Beta vulgaris* L.) is highly consumed, but its stems and leaves are often discarded. This work aims to characterize the chemical properties and bioactive compounds in beet stems and leaves and assess their applicability in food products. The stems and leaves were subjected to different drying temperatures (50 to 70 °C) to determine the optimal temperature for preserving their bioactive compounds. They are then nutritionally and physiochemically characterized and incorporated into a food matrix. The optimal drying temperature was 60 °C. The leaves and stems contain approximately 30 and 15 g/100 g of protein, 30 and 32 g/100 g of dietary fiber, 4 and 0.45 g/100 g of lipids, and 24 and 25 g/100 g of ash, respectively. Both provide approximately 50% of the amino acid requirements established by the WHO/FAO/UNU and are rich in iron and potassium. The stems presented 53% more betalainic compounds (0.58 mg/g) and a higher nitrate content (359 mg/kg) than did the leaves, which presented a higher polyphenol content. The incorporation of flour from beet stems and leaves into food products is economical, reduces food waste, and enhances nutrition and health.

## 1. Introduction

In 2015, world leaders adopted a set of global measures to eradicate poverty, protect the planet, and ensure prosperity for all as part of a strategy for sustainable development [1]. In this context, Goal 12, responsible production and consumption, relies on the appropriate use of the environment and resources in a sustainable manner. In this sense, a global issue is the waste produced by the food industry, mainly in the processing of fruits and vegetables. The United Nations estimated that global food loss is approximately 1.6 billion tons per year, resulting in waste [2]. According to this report, it is estimated that the percentage of food losses and waste produced by fruits and vegetables ranks first, followed by oilseeds and pulses at approximately 20% and 10% in Europe and North and Latin America, respectively, whereas in Asia and Africa it reaches values of less than 15% and 10%, respectively. Food waste not only wastes resources such as water, land, and energy, leading to environmental pollution, but also results in significant losses during production and processing, with up to 45% of raw materials wasted. However, these food industry by-products are valuable sources of bioactive compounds and natural pigments that can be repurposed to create functional foods. Food waste and by-products utilization can be divided into three categories: reuse, recycling and upcycling/valorice, and the latter converts the food waste and by-products into higher-value products. In this context, the greatest amount of food waste is produced by the fruit and vegetable processing industry [3].

Beetroot (*B. vulgaris* L.) belongs to the Chenopodiaceae family, with more than 1400 species [4]. This plant has three main organs: the root, neck, and leaves. The root is conical in shape and rarely protrudes from the ground. Inside this root, a series of alternating light and dark concentric rings are distinguished. The lighter ones stand out for having softer tissue but are rich in sugar, whereas the darker ones consist of fibrous tissues formed by bundles of vessels that extend to the juice of the root leaves [5]. Beetroot and its bioactive compounds, including nitrates and betalains, have attracted significant interest due to their reported health benefits for both human health and the food industry [6,7,8].

After a series of reactions in the body, nitrates transform into nitric oxide (NO), which acts as a vasodilator in the arteries, thereby improving blood flow and inhibiting platelet aggregation. Some benefits of this component are attributed to its ability to control hypertension, and it is used as part of pharmacological treatment [9]. In sports nutrition, this compound has been studied in detail, reporting natural ergogenic properties that improve performance in athletes. Betalains, which are responsible for producing beetroot color, are compounds derived from tyrosine synthesized from the nitrogen core structure of betalamic acid [10]. In the “Betalain profile, content and antioxidant capacity of red beetroot dependent on genotype and root part” study, the authors reported that 30 different betalains are produced in beetroot tissues, constituting 18 betacyanins and 12 betaxanthins, with each type having specific characteristics and correlating with the antioxidant activity of beetroot [11]. The structural components of betalains (a phenolic amine and a cyclic amine) are good electron donors, so betalains are considered to have antioxidant properties [8]. Other benefits attributed to betalains for human health are their anticancer properties, even if they are patented in the United States as components of cancer drugs [7]. These properties are based on the cytotoxicity of betalains and their associated plant extracts containing them to cancer cell lines. These compounds also have anti-lipidemic effects. In a study where volunteers consumed 250 g of edible pulp, total cholesterol and LDL cholesterol levels were reduced [8,12].

In the food industry, betalains are used as colorants, meeting the current consumer requirements for natural food. Red beetroot betanin is a common red dye and is the only compound approved for use by the European Union and the United States. Its main advantage is that it is not only a colorant but also an antioxidant, which implies that it has no basic contraindications for human health [8]. Other studies have shown that beetroot has antimicrobial activity, inhibiting the action and multiplication of some bacteria, such as Salmonella typhimurium. This activity seems to affect the structure, permeability, and other functions of the microorganisms’ cell membranes, leading to cell death [13]

Beetroots are mainly used for human consumption because of their recognized nutritional and health benefits, and their demand has increased in recent years. Originally, beetroots were grown for the consumption of their leaves, but over the years, the main edible product has become the bulb, and the leaves and stems are discarded as waste and remain unexploited. From a nutritional point of view, several studies have highlighted their rich and varied nutritional composition, and many authors emphasize not only the bulb but also the particular nutritional content of the leaves and stems [14]. For proteins, a protein content of 1.61% was reported in beetroot bulbs and 22.8% was reported in the leaves.

Beetroot leaves and stems are usually considered compost, animal feed, or food waste [15]. In recent years, a significant number of research studies have demonstrated that waste or by-products from this type of industry are promising sources of high-value compounds with antioxidant and/or antimicrobial properties, making them potential raw materials for component extraction [16].

Various studies have highlighted that the leaves of this vegetable are an excellent source of bioactive compounds, such as polyphenols and pigments (betalains), which confer a high antioxidant capacity to the material [17].

In 2014, Biondo conducted a nutritional characterization of beetroot leaves at different developmental stages, highlighting their antioxidant activity and the potential benefits of using them as food for human nutrition [18]. In view of the above, this work aims to characterize the chemical properties and bioactive compounds present in beet stems and leaves and assess their applicability in food products.

## 2. Materials and Methods

### 2.1. Materials

The beetroot leaves and stems were obtained from Dos Marías Farm, an agricultural company located in the Coquimbo Region, Chile. The plants were collected after approximately 60 days after cultivation and with an Ecuadorian diameter of approximately 5–10 cm diameter at the beetroot bulb consumption maturity stage. They were carefully selected, fresh, of green color, avoiding dry or yellow leaves, with undamaged leaves and/or stems, whole, and without any signs of mechanical or microbiological deterioration. After the leaves and stems were collected, both were washed with tap water, and the excess water was removed and subsequently stored at 4 °C until further analysis.

### 2.2. Methods

#### 2.2.1. Sample Preparation and Drying Process

Prior to the drying process, the leaves and stems were disinfected with 40 ppm sodium hypochlorite for 10 min, then were rinsed, and the excess water was removed before being cut into small pieces. For meal generation, beetroot by-products were dried in an oven Biobase Model BOV-V70F, Biobase, Shandong, China at temperatures ranging from 50 to 70 °C until they reached an approximate moisture content ranging from 7 to 10% based on previous work by Asadi and Khan [19]. The dried samples were subsequently milled in a micropulverizer RETSCH model ZM-200 (RETSCH, Haan, Germany) at 10,000 rpm. The milling conditions were selected on the basis of the particle size of the flour, which should be similar to that of conventional flour, and then the flour was sieved.

During the drying process, the sampling mass at time t (*Mt*) was recorded at 30 min intervals. The moisture ratio (*MR*) corresponds to the relationship between the mass of the sample at different times and the moisture at each time. *MR* was calculated via Equation (1):

(1) $$MR=\frac{Mt-Me}{M_{0}-Me}$$

where *Mt* is the moisture content at time = *t*, *Me* is the equilibrium moisture content, and *M*_0_ is the initial moisture content [20].

#### 2.2.2. Proximate Chemical Characterization and Water Activity

The proximate composition was determined in triplicate via the American Association of Cereal Chemists AACC method, and the results are reported as g/100 g on a dry basis [21]. The moisture content was determined in an oven Biobase Model BOV-V70F, Biobase, Shandong, China at 105 °C until a constant weight was reached; the protein content was estimated by using the Kjeldahl technique (nitrogen conversion factor of 6.25); the lipid fraction was extracted with hexane under reflux conditions by the Soxhlet technique in Soxtec 2050, FOSS, Labexchange, Swabian Alb, Germany; the ash content was obtained by incineration in a muffle furnace at 600 °C according to the official Methods 08-03 [22]; and the total (TDF), soluble (SDF), and insoluble (IDF) dietary fiber contents were determined by the total dietary fiber assay procedure of AOAC Method 991.43, which is based on an enzymatic and gravimetric method [23]. The water activity (aw) was analyzed by a dew point water activity meter (AquaLab 4 TE, Pullma, Washington, DC, USA, at 25 ± 0.5 °C).

#### 2.2.3. Amino Acid Profile

For the determination of the amino acid profile, the PICO-TAG method by Waters Associates was utilized [24]. This technique involves amino acid derivatization prior to column separation using phenyl isothiocyanate (PITC). This procedure consists of 3 steps: (i) sample hydrolysis with 6 N hydrochloric acid containing 0.1% phenol at 110 °C for 20 h to produce free amino acids, (ii) precolumn derivatization of the samples with PITC, and (iii) analysis by reverse-phase high-performance liquid chromatography (HPLC). Chromatographic separation of the hydrolysates was performed using a reverse-phase Pico-Tag column (3.9 × 300 mm) with a C18 column at 400 °C and a UV detector at 254 nm.

The mobile phase consisted of 2 eluents, an aqueous buffer (A) and 60% acetonitrile in water (B). The aminoacid derivatives were separated by gradient of eluent A and eluent B at 1 mL/min. The separation gradient ranged from 10% B to 51% B over 10 min. Amino acid identification in the samples was achieved by comparing the retention times with those of standards.

#### 2.2.4. Fatty Acid Profile

For the identification of the fatty acid profile, a methodology based on GC-FID according to AOCS/CE-1J07; AOAC 969.33 was used. Five grams of dried sample was weighed, and 20 mL of hexane was added. The mixture was agitated for 2 h and left to settle for 24 h. Afterward, the solution was filtered and transferred to a rotary evaporator for an additional 24 h. Three drops of the obtained material were taken, and 3 mL of heptane was added. The mixture was vortexed for 30 s, followed by the addition of 200 µL of alcoholic KOH (methanol). The mixture was vortexed for 30 s and left to settle for 30 s until 2 phases were clearly observed. It was then injected at 1 mL/min into an Agilent Technologies 689 N Network gas chromatograph at a gradient temperature from 120 °C to 230 °C equipped with an autosampler and a flame ionization detector (FID). The system was equipped with a 60 long SP-2330 silica capillary column. Fatty acid identification was carried out by comparing the retention times with patterns of methyl esters of fatty acids.

#### 2.2.5. Mineral Composition and Phytic Acid Determination

Minerals were measured with a flame atomic absorption spectrometer at the Analysis of Soils, Plants and Water Service at Institute of Agricultural Sciences, Madrid (Spain).

The samples were previously digested by means of HNO_3_ and H_2_O_2_ attack by irradiation at 800 W (15 min at 180 °C) in a microwave-accelerated reaction system (MARS, Charlotte, NC, USA). Phytic acid (myo-inositol 1,2,3,4,5,6-hexakisphosphate, InsP6) was measured as phosphorus released by the action of phytase and alkaline phosphatase by a spectrophotometric method with a commercial kit (K-Phyt 07/11 Megazyme, Bray, Ireland). The samples were analyzed in triplicate.

#### 2.2.6. Functional Properties and Color

The functional properties were measured in triplicate. The solubility of each sample was determined by using a solution of 1% *w*/*w* at 30, 60, 70, and 80 °C according to the methodology of Vera, Laguna [25]; the water absorption capacity (WAbC) was determined by the difference in weight according to the AACC method 88-04 [26]; the water adsorption capacity (WAdC) was determined by using the method described by Segura-Campos, Ciau-Solis [27], where the samples were exposed to 98% relative humidity provided by the potassium sulfate solution and saturated until a constant weight was reached; and the water and oil holding capacities (WHC/OHC) were determined by difference in weight once the samples reached their complete hydration, according to the method described by Timilsena, Adhikari [28]. The color of the stem and leaf meals were determined by using a CN-200 colorimeter, which has a CIE 10° standard observer and CIE D65 illuminant. The results are expressed in CIELab coordinates: L* (lightness), a* (red to green), and b* (yellow to blue). The color difference values (Δ*E*) were calculated according to Equation (2).

(2) $$∆E={\surd (∆L)}^{2}+{\left(∆a\right)}^{2\;}+{\left(∆b\right)}^{2}$$

where Δ*L*, Δ*a*, and Δ*b* are the differences between the L* values, a*, and b*, respectively.

#### 2.2.7. Extraction of Bioactive Compounds

For the determination of betalainic pigments, total polyphenols and antioxidant capacity, the method proposed by Ravichandran, Saw [29] was used. Fifty milligrams of dried beetroot leaves or stems were added to 1 mL of an extractant solution of water/methanol/formic acid (84.95/15/0.05) and heated at 60 °C. The mixture was shaken on a vortex for 30 s and then in an ultrasonic bath for 30 s and finally centrifuged for 15 min. The obtained supernatant was extracted, and the process was repeated 4 more times on the residue. The obtained extracts were centrifuged once more and brought to a final volume of 5.0 mL before use.

#### 2.2.8. Determination of Betalains, Total Phenolics, and Antioxidant Capacity

The betalain content was determined by using a UV–VIS spectrophotometer (Shimadzu, model UV-1900) according to the methodology proposed by Ravichandran, Saw [29]. The contents of the betacyanins and betaxanthins in the extracts were determined by measuring the absorbance at two wavelengths, 538 and 480 nm, respectively. To determine the concentration of betalains, Equation (3) was used:

(3) $$B=\frac{\left(A\times DF\times MW\times 1000\right)}{\varepsilon \times L}$$

where *A* is the absorbance of the sample, *DF* is the dilution factor, *L* is the length of the optical path of the cuvette, *MW* is the molar mass of the compound, and *ε* is the molar extinction coefficient. The molar mass and extinction coefficient used were 550 g/mol and 60,000 L/mol cm in water for betacyanins and 308 g/mol and 48,000 L/mol cm in water for betaxanthins. The measurements were performed in triplicate, and the results are expressed in mg/g dry basis (d.b.)

The extracts obtained in Section 2.2.7 were subjected to total polyphenol content (TPC) analysis. The TPC was measured by using the Folin–Ciocalteu method [30] and the absorbance was measured with a UV–VIS spectrophotometer Shimadzu, model UV-1900, Shimadzu, Kioto, Japon at a wavelength of 725 nm. A standard gallic acid calibration curve was used for quantification. The results are expressed in mg of gallic acid equivalents per 100 g of dry basis.

The extracts obtained in 1.2.5 were analyzed by a DPPH (2,2–diphenyl–1–picryl–hydrazyl) assay to determine the antioxidant capacity. The analysis was performed according to the method developed by Brand-Williams, Cuvelier [31]. In cryotubes, 100 µL of extract was mixed with 2.9 mL of DPPH (80 mg/L in 80% methanol) and left in the dark for 30 min. The absorbance at 517 nm was subsequently determined with a UV–VIS spectrophotometer Shimadzu, model UV-1900, Shimadzu, Kioto, Japon. It was quantified using a Trolox calibration curve, and the values are expressed as Trolox equivalent (µmol ET/100 g dry basis).

#### 2.2.9. Determination of Nitrate

For nitrate determination, a liquid chromatography system based on the AOAC 935.48 method was used. An HPLC C18 column was used for separation. The samples were homogenized beforehand. One gram of sample was weighed for each sample. The mixture was subsequently placed in a flask in a boiling water bath for 20 min, agitated at 80 °C, and left to cool before it was finally diluted in 100 mL of deionized water. The mixture was subsequently filtered through a 0.45 µm syringe filter. All the samples were analyzed immediately after 1 h of sample preparation. They were then passed through the mobile phase through the HPLC column. The flow rate was 0.8 mL/min, and the detection wavelength was 213 nm. The peaks of the samples were identified by comparison with the nitrate standard.

#### 2.2.10. Food Product Development and Organoleptic Analysis

For mineral determination and snack preparation, a drying temperature of 60 °C was selected, considering other studies conducted on beetroot discards at this temperature [18,19]. This temperature is suitable for assessing the behavior of the minerals under study under conditions representative of certain processes or conditions in product development and its potential influence on the organoleptic characteristics it could impart to the product.

After drying, the flour was ground and sifted to achieve a particle size similar to that of regular wheat flour. A tortilla chip-type snack was made from corn flour and beetroot stem and leaf flour in different proportions. One batch was prepared as a control, in which only corn flour was used, while the other batches included waste flour. Table 1 presents the quantities used for each tortilla chip.

The sensory evaluation of the tortilla chips was initially conducted through sensory tests using initially a panel of 41 semi-trained individuals (4 males and 37 females) ranging from 20 to 26 years old. The members of this panel were students from the Nutrition and Dietetics Program in the Coquimbo Region. Prior to starting, they were informed about potential food allergies and signed an informed consent form to participate in the research. Threshold tests were conducted to determine the sensitivity to the four basic tastes (sweet, salty, sour, and bitter), as well as tests for odor identification and evaluation of acidity and sweetness intensity. The training was carried out for two weeks. The panelists who achieved an approval rate exceeding 50% of recognition of basic tastes and odor were selected to participate in the sensory panel according to the semi-trained definition by Lawless and Heymann [32].

The sensory evaluation of the tortilla chips was performed using a 5-point hedonic scale, where the tortilla chips were rated from 1 to 5 according to their level of preference. Organoleptic evaluation was performed for one day to ensure standardized conditions. The panelists were asked to evaluate various samples on the basis of different sensory attributes, including their color, flavor, texture, and general acceptability.

#### 2.2.11. Statistical Analysis

The analyses were performed in triplicate and the results were expressed as mean value ± standard deviation. One-way ANOVA was used to compare means values, and significant differences (*p* < 0.05) were calculated with Tukey’s post hoc test. All tests were performed using the software Statgraphics Centurion XVI.

## 3. Results

### 3.1. Drying Characteristics

**Figure 1:**
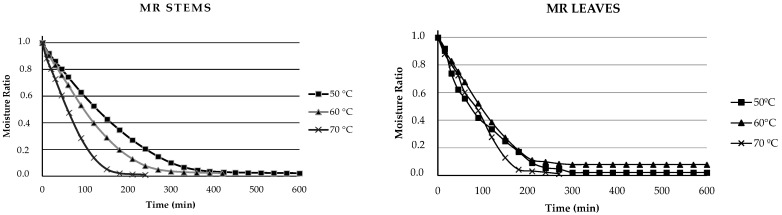
Effects of drying temperature on the moisture content curves of beetroot by-products over time. The values are means of triplicate analyses (*n* = 3) and the error bars represent the standard deviation.

The drying curves are presented in Figure 1. To analyze the time/moisture relationship, the “moisture ratio” (MR), which is related to the necessary drying time to reduce moisture, was used. This allowed us to measure the efficiency of the drying process and determine how much time is needed to remove moisture from beetroot discards [33]. The results indicate that drying time decreases as the temperature increases, both for leaves and stems. Elevated temperatures typically accelerate evaporation and moisture removal processes as expected [33]. The difference in drying time between the leaves and stems can be attributed to the structural characteristics and nutritional composition of each sample. In general, a higher density is related to slower heat transfer and reduced moisture evaporation. In this study, leaves are thinner and thus have more exposed surfaces than denser stems do, which contain more structural tissue or compact structures [34]. These differences in density affect the drying kinetics. In addition, leaves dried at 60 °C require a drying time of 240 min to reach equilibrium compared with the 180 min required for leaves dried at 70 °C. In the case of stems, this time trend was repeated, requiring 450 min for drying at 50 °C and 360 min for drying at 70 °C. Currently, drying processes that have a lower impact on energy consumption to reach equilibrium are being sought, considering the importance of seeking processes that are more sustainable in the use of resources [35].

### 3.2. Proximate Chemical Composition and Water Activity

For several years, unconventional food plants (UFPs) have been investigated as emerging candidates for the development of new food matrices with better nutritional compositions [36]. In this context, the stems and leaves of *B. vulgaris* L. are excellent nontraditional alternative sources of nutrients.

The results for the proximate composition of meals from beet stems and leaves dried at 50, 60, and 70 °C expressed on a dry basis are presented in Table 2. In terms of moisture content and water activity, the samples dried at a temperature of 50 °C presented a moisture content of 3.44% and 8.49% for the leaves and stems, respectively. This difference can be explained by the structure of the leaves, which are thinner and have more readily available free water, and by the differences in nutritional composition [34].

In general, all the samples had high protein and ash contents. Notably, the protein content of the leaves was greater than the stems, reaching values greater than 30 g/100 g and ~15 g/100 g, respectively. These results were higher than those by Tamayo Tenorio, Schreuders [37] who reported values for leaves between 19.4 and 22.8 g/100 g on a dry basis and higher than those reported by Akyüz and Ersus [38] for leaves on a dry basis (24.02 ± 0.18 g/100 g). These results were lower than those previously reported by Abdo, El-Sohaimy [39], who detected 27.22 g/100 g d.b. of proteins in stems and 60.65 g/100 g d.b. of proteins in leaves. Moreover, the results of the present investigation are also greater than those reported by Kiskini, Vissers [40] for beet leaves at different developmental stages. These differences can be attributed to the different varieties used for each study, as well as the growth conditions and the environmental and agronomic characteristics, but not on the plant age, which is in agreement with the findings reported by Kiskini, Vissers [40]. The values obtained in this study exceed those of many plant products; for example, according to the USDA database, quinoa, amaranth, and chia seeds have approximately 16, 15, and 18 g/100 g protein, respectively [41]. Compared with other leaves and roots, in a recent study by Waseem, Akhtar [42], dehydrated spinach produced 19.2 g/100 g of protein, whereas beetroot powder produces 13.5 g/100 g of protein [43]. In both stems and leaves, temperature did not significantly influence the protein content.

On the other hand, ash represents the food’s mineral content; in this case, both stems and leaves have a high mineral content, ranging between 24.88 and 25.47 g/100 g and between 14.20 and 15.24 g/100 g, respectively. This high mineral content has been previously reported by Fernandez, Jagus [17], who studied beet greens; by Abdo, El-Sohaimy [39], who analyzed beetroot leaves and stems; and by Asadi and Khan [19], who investigated dried beetroot leaf powder. On the other hand, significantly greater amounts of total dietary fiber were found in leaves and stems, with no significant differences among the temperatures (Table 2). Similar results were reported by Asadi and Khan [19] for dried beetroot powder. In addition, this high amount of dietary fiber has also been reported [17]. The consumption of a substantial amount of dietary fiber lowers the risk of developing several conditions, including coronary heart disease, stroke, hypertension, diabetes, obesity, and certain gastrointestinal disorders [44].

Finally, all samples, both leaves and stems, reached equilibrium and showed values of water activity less than 0.5. It has been reported that environments with low water activity in foods, generated by food preservation processes such as drying, lead to the inactivation of microorganisms, thereby increasing shelf life. These desirable effects are achieved at levels below 0.6 [45].

### 3.3. Amino Acid Profile

The significant protein levels contained in stems and mainly in leaves make them appealing from a nutritional perspective (Table 2). The health benefits provided by plant-based proteins rely on their biological value, which is determined by the presence of essential amino acids. In this context, Table 3 presents the amino acid compositions of leaves and stems from beetroot, both essential and non-essential, that were dried at different temperatures.

According to the results, temperature affects the amino acid composition, i.e., as the temperature increases from 50 to 70 °C, the amount of each amino acid decreases, which is clearly observed for essential amino acids (EAAs).

**Figure 2:**
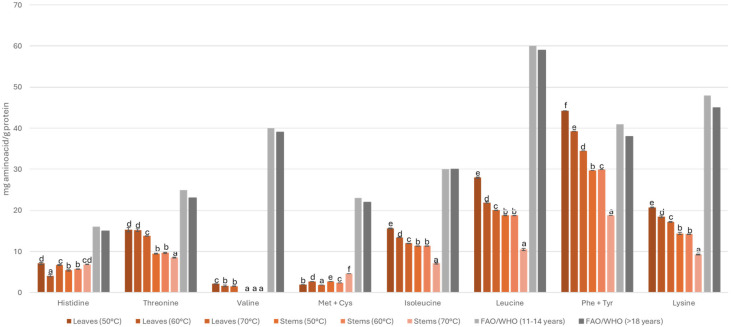
Composition of amino acids (mg/g protein) based on the FAO/WHO/UNU (Food and Agriculture Organization/World Health Organization/United Nations University) standard reference pattern (g/100 g protein). The values are expressed as mean ± standard deviation (*n* = 3). Different lowercase letters indicate significant differences according to Tukey’s multiple range test (*p* < 0.05).

Figure 2 shows the composition of amino acids based on the standard reference patterns of the FAO/WHO/UNU (Food and Agriculture Organization/World Health Organization/United Nations University) [46]. The amino acid content between stems and leaves varies considerably, while the leaves have all the essential amino acids, the stems are deficient in valine.

In terms of specific amino acids, for histidine, both the leaves and stems dried at any of the temperatures used in the study provided approximately 50% of the requirements. In terms of threonine, leaves provided about 67% and stems about 42% of the requirements, while leaves dried at 50 °C provide 52% of isoleucine and 54% of leucine. In addition, leaves dried at any temperature can provide the 46% of lysine established by the WHO/FAO/UNU.

Finally, leaves dried at 50 °C exceeded the FAO/WHO reference values of phenylalanine plus tyrosine. The amino acid profile of the leaves makes them attractive from a nutritional point of view.

### 3.4. Fatty Acid Profile

Fatty acids play a crucial role in a variety of biological functions, as they are essential components of cell membranes and participate in the regulation of inflammatory processes, as well as in maintaining cellular integrity, directly impacting health statuses [47]. In the specific context of beetroot by-products, the fatty acids profile is presented in Table 4, detailing the total content of saturated fatty acids (SFAs), monounsaturated fatty acids (MUFAs), and polyunsaturated fatty acids (PUFAs). The amount of fatty acids was greater in the leaves than in the stems. These differences in fatty acids present in the leaves compared with the stems could be attributed to the presence of lipids contained in the chloroplasts as previously reported by Biondo, Boeing [18]. Plants can remodel their membranes and synthesize a greater amount of fatty acids for the assembly of glycerolipids in the membranes of chloroplasts and the endoplasmic reticulum as previously described by Cook, Lupette [48]. In the case of leaves, significant differences were detected among the three temperatures evaluated, with their content decreasing with increasing temperature. Among the fatty acids present in the leaves, palmitic acid stands out for its significant ability to prevent cardiovascular diseases [49]. Omega-3 fatty acids also stand out in the leaves and are among the main fatty acids present in the leaves. This finding is relevant in the search for plant-based foods that provide omega fatty acids, especially in the context of vegan diets [50]. The LA/ALA ratio increased with increasing drying temperature both in the leaves and in the stems. This ratio is crucial for assessing the nutritional quality of food, suggesting that drying at lower temperatures could preserve a more balanced fatty acid profile [51].

### 3.5. Determination of Mineral Composition and Phytic Acid

The mineral composition and phytic acid content are shown in Table 5. According to the USDA Food Database, the minerals found mostly in these by-products are calcium, potassium, and magnesium, which makes them excellent sources of minerals [41].

For the mineral content analysis, a temperature of 60 °C was used because it was the temperature selected to evaluate the potential impact on snack preparation. The analysis revealed a high content of potassium and magnesium in stems and a high content of calcium and potassium in leaves, which is consistent with reports in beetroot (*B. vulgaris*) compositions by Abdo, El-Sohaimy [39]. Considering that these minerals help control blood pressure with consistent consumption, these findings suggest that the discards could be a beneficial source for preventing high blood pressure [52]. Additionally, the nitrate content known for its vasodilatory properties and potent hypotensive effects further supports its potential application [53]. Interestingly, the potassium concentration in the stems was double that in the leaves. In addition, a significant amount of iron is present in the leaves and in stems. According to Khare, Samudre [54], iron is one of the most important minerals that contributes to the treatment of anemia. In this context, the results revealed 43.8 mg/100 g in the leaves and 15.95 mg/100 g in the stems, which exceeds the recommended dietary allowance (DRI) of iron of approximately 7–11 mg/day [55,56]. However, studies have highlighted the amount of this mineral in beetroot discards.

**Table 5:** Mineral composition.

		Minerals	Units			Stems				Leaves	
		Ca		mg/100 g		325.00	±	21.21 ^a^	1445.00	±	7.07 ^b^
		Mg		mg/100 g		590.00	±	28.28 ^a^	1310.00	±	28.28 ^b^
Macroelements		P		mg/100 g		315.00	±	7.07 ^a^	300.00	±	14.14 ^a^
		K		mg/100 g		6155.00	±	120.21 ^b^	3040.00	±	0.00 ^a^
		Cu		mg/100 g		0.45	±	0.07 ^a^	1.15	±	0.07 ^b^
		Mn		mg/100 g		5.65	±	0.64 ^a^	33.85	±	3.61 ^b^
Microelements		Zn		mg/100 g		1.85	±	0.07 ^a^	2.75	±	0.07 ^b^
		Fe		mg/100 g		15.95	±	0.92 ^a^	43.80	±	1.13 ^b^
	Flour (g) *					1	5	10	1	5	10
	Ca		DRIs 1000		mg/day	33	163	325	145	723	1445
	Mg		DRIs 350/300		mg/day	169/197	843/983	1689/1967	374/437	1871/2183	3743/4367
Contribution to DRIs (%) *	P		DRIs 550		mg/day	57	286	573	55	273	545
	Cu		DRIs 1.6/1.3		mg/day	28/35	141/173	281/346	72/88	359/442	719/885
	Mn		DRIs 2.3/1.8		mg/day	246/314	1228/1569	2457/3139	1472/1881	7359/9403	14717
	Zn		DRIs 11/8		mg/day	17/23	9.25	18.5	2.75	13.75	18806
	Fe		DRIs 11/7		mg/day	145/228	725/1139	1450/2279	398/626	1991/3129	3982/6257
Ratio	Ins P_6_/Ca < 0.24						21.50			6.68	
	Ins P_6_/Fe < 1						0.0006			0.0003	
	Ins P_6_/Zn < 15						0.006			0.006	

Values followed by the same letter in the same row are not significantly different (*p* < 0.05). InsP6—Myo-inositol phosphate. DRIs—dietary reference intakes: recommended dietary allowances and adequate intake elements (male/female). Life stage group: >18 years; [53].* Contribution based on snack formulation in 2.2.10. (1, 5, and 10 g/100 g model food).

Phytic acid (inositol hexaphosphate) is a molecule that contains phosphate and is used by plants to allow the independent growth of seeds through its use as a source of nutrients [57]. On the other hand, phytic acid also has the capacity to affect the solubility of divalent ions such as zinc and Fe because of its double-charged phosphate groups [58].

Due to the high amount of minerals present in leaves and stems, phytic acid was used to estimate mineral availability. The World Health Organization presented several criteria for zinc and acid phytic proportions, with a phytate–zinc < 5 ratio, indicating a negative impact on the absorption of zinc [59]. In the case of iron, the suggested acid phytate–iron ratio is <1 and the pytate–calcium ratio is <0.24 according to Hurrell [60]. The inhibition of iron or zinc absorption is related to the level of phytate in a food [59]. The mechanism of this inhibition has been reported by Marolt, Gričar [61] and is explained as the formation of metal insoluble complexes with phytic acid, which depends mainly on the molar ratio, protonation state related to the pH level and side reactions. The acid phytic–zinc ratios of both the leaves and stems were lower than 0.006, suggesting that the phytic acid content should not interfere with zinc absorption. On the other hand, the acid phytic–iron ratios of both the leaves and stems were 0.0003 and 0.0006, respectively, which suggest that there was no interference in iron absorption. In addition to iron and zinc, the acid phytic–calcium ratios of both the leaves and stems were 6.8 × 10^−6^ and 2.2 × 10^−5^, respectively, which indicates that the phytic acid does not interfere with calcium absorption.

In terms of the dietary reference intakes (DRIs), mineral absorption inhibitors are absent. Table 5 shows the contribution to the DRIs of dried leaves and stems at 60 °C expressed in % and calculated on the basis of an intake of 1; 5, and 10 g/day (on the basis of the formulation in Section 2.2.10). In this context, when 1 g/day of leaves is considered, the DRIs for calcium and magnesium exceed 100% of the requirements; while for stems, only magnesium exceeds the requirement. On the other hand, considering 5 or 10 g/day, all the DRIs for all minerals exceed 100% of the requirement.

### 3.6. Functional Properties and Color

The functional properties are presented in Table 6. The water adsorption capacity (WA_d_C) reflects how much of the water in the environment can be retained on the surface of a sample. According to the results, the drying temperature had no influence on the WA_d_C in either the leaves or the stems, but the WA_d_C in the stems was almost double that in the leaves. On the other hand, stems showed greater water absorption capacity (WA_b_C) than did leaves, and the WA_b_C was greater at 50 °C for both leaves and stems. In terms of water holding capacity (WHC), the WHC of the stems was greater than that of the leaves, which could be attributed to the greater amount of dietary fiber found in the stems. On the other hand, while the WHC of the leaves did not significantly differ among the temperatures, on the stems, the WHC decreased with increasing temperature. The oil holding capacity did not differ between the stems and leaves, and the temperature did not influence the capacity. Different WHC and OHC values, 2.00 and 3.56 g of water or oil/g sample of WHC and OHC, respectively, were reported by Mitrevski, Pantelić [62] for beetroot powder. Some functional properties of beetroot leaf powder dried at 60 ± 2 °C for 7 h were analyzed by Asadi and Khan [19], and the results revealed a lower WA_b_C and OHC than those reported in the present study.

In general, the solubility pattern is different between leaves and stems, with higher solubility observed in stems. The solubility ranged from 33 to 42% for leaves and 45 to 49% for stems. The stem solubility increased slightly with increasing temperature, whereas the leaf solubility decreased with increasing temperature. Solubility has a strong effect on the functionality and relative stability of the ordered and disordered structures of polysaccharides in solution, determining whether they will be completely or partially dissolved or whether they can be solubilized [63].

The colors of the leaves and stems dried at the three different temperatures are shown in Table 7. The color of both the leaves and stems changed with increasing thermal treatment. In the case of the leaves, the samples became darker and more yellow at higher temperatures. These changes in color could be attributed to the Maillard reaction or caramelization of sugars, whereas the green color observed in stems could indicate changes in chlorophyll concentration [64]. On the other hand, stems wither and become greener at higher temperatures. The color of beetroot leaves was also reported by Asadi and Khan [19], who reported differences in L*, a*, and b* but with similar trends. These differences could be attributed to different factors, such as the maturity stage of the plant parts and the environmental and agronomic conditions.

### 3.7. Determination of the Phenolic Content, Total Phenolic Content, and Antioxidant Capacity

Betalains are nitrogenous compounds responsible for the coloration of beets. There are two types: betacyanins, which are responsible for red and violet colors, and betaxanthins, which are responsible for yellow colors [65]. However, their main beneficial characteristics are related to phenolic compounds, providing them with antioxidant properties [66].

**Figure 3:**
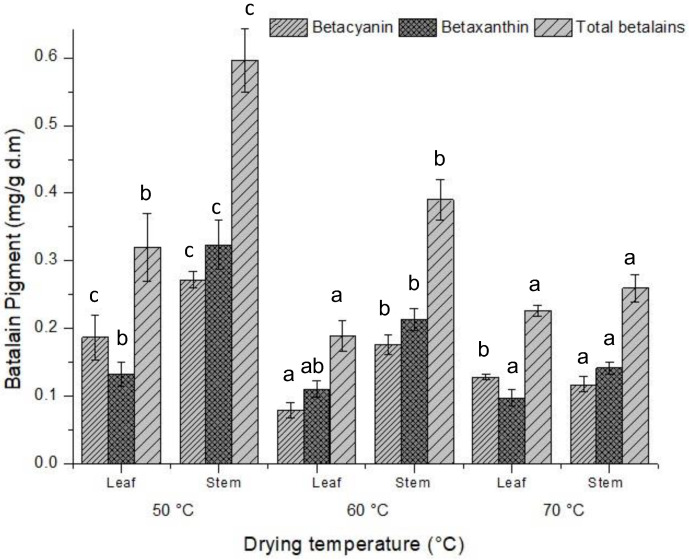
Effect of drying temperature on betalain content. Values are averages (*n* = 3). Different lowercase letters indicate significant differences according to Tukey’s multiple range test (*p* < 0.05).

In this study, the stabilities of total betalains, betacyanins, and betaxanthins dried at different temperatures were evaluated. Figure 3 shows that the samples that retained the greatest amount of these compounds were those dried at 50 *°C*, both in the stems and leaves. For total betalains, there were significant differences among the three temperatures in the case of the stems. For the leaves, the differences were focused on the samples dried at 50 °C, with no significant differences between the other two temperatures. This decrease is repeated for both betacyanins and betaxanthins.

In terms of total betalainic compounds in the stems and leaves, the results revealed greater amounts in the stems, with 53% more betalainic compounds than in the leaves. This trend is consistent with those reported by Ben Haj Koubaier, Snoussi [67], who studied the betalain content, phenolic composition, and antioxidant activity of different parts of beetroot. The color change is related to the degradation of betalains, as reported by de França, Cruz-Tirado [68]. In our study, betalainic compounds also degraded at relatively high temperatures, explaining the labile characteristics of these compounds. These data coincide with those reported by Shankar Bunkar, Anand [69], where betalainic compounds decreased with increasing temperature with convective drying.

The results of total polyphenol content (TPC) of beet leaves and stems are detailed in Table 8. There is an appreciable content of polyphenolic compounds, with leaves consistently showing higher TPC than stems. Specifically, both the leaves and stems of plants dried at 50 °C exhibited greater TPC than did those of plants dried at 60 °C or 70 °C. This trend indicates a gradual decrease in the TPC as the drying temperature increases. However, no significant differences are observed between the results obtained at the different drying temperatures. These results are consistent with those reported by Ben Haj Koubaier, Snoussi [67], who noted variability in polyphenol content among different beet parts in terms of antioxidant activity. Both the leaves and stems tended to exhibit decreasing antioxidant activity as the drying temperature increased. This can be explained by the effect of temperature on polyphenolic compounds, which are affected by increasing temperature and are directly related to the total betalain and total polyphenol contents, which are responsible for this antioxidant activity.

### 3.8. Analysis of Nitrates

Nitrates are compounds primarily found in leafy greens, roots, and tubers and to a lesser extent in fruits and legumes [1]. Numerous studies have focused on the benefits of nitrates, with their vasodilatory properties being a major contributor to health and nutrition. One of the foods that has been studied in relation to the benefits of nitrates in the human diet is beetroot, although to date, the quantity of nitrates present in its stems and leaves has not been investigated. This study aimed to quantify this compound in these by-products, highlighting the greater concentration of nitrates in the stems than in the leaves. The nitrate data are presented in Table 8. The drying temperature of 50 °C resulted in the greatest amount of nitrates, with a total of 359 mg/kg in the stems. In the case of the leaves, the drying temperature of 50 °C also yielded the highest nitrate concentration, with a total of 159 mg/kg, which was significantly different from that of the other drying temperatures. In a study conducted by Benjamim, Sousa [1], different vegetables were classified into three categories based on their nitrate content: high, medium, and low. The beet stems were considered medium nitrate-contributing vegetables, with nitrate levels ranging from 168 to 518 mg/kg. On the other hand, leaves are categorized as low nitrate-contributing vegetables, with values ranging from 25 to 203 mg/kg. Notably, the drying temperature of 70 °C resulted in the lowest content (100 mg/kg), whereas the 50 °C drying temperature resulted in the highest value of 159 mg/kg. The recommendations for nitrate consumption vary depending on various studies and factors, such as the type of exercise performed. According to the research by Benjamim, Sousa [1], which was based on experimental studies involving individuals’ recommendations for the use of nitrates as ergogenic aids, ranges from >300 mg when chronic consumption is suggested to 800 mg when acute effects are active after exercise [70]. In the case of beetroot by-products, stems dried at 50 °C and 60 °C fully meet these recommendations for chronic use as ergogenic aids.

### 3.9. Organoleptic Analysis

The sensory attributes of the beetroot by-products snacks are presented in Table 9. Compared with those of the control sample, the texture attributes of the samples made with only corn flour were not significantly different.

The color attribute measured by the panel of semi-trained individuals exhibited the most significant differences, as expected, which were in agreement with the color results obtained in Table 6, where b* of the leaves had the highest value. The flavor of snacks made with 10% beetroot waste flour was better evaluated than the control snacks. The overall acceptability of the snacks was not significantly different between the control group and the 10% beetroot waste flour group. The snacks made with 20% beetroot waste flour had lower acceptability both overall and for each of their attributes, although none of the snacks scored below three. This decrease in overall acceptability may be due to the properties of betalains, which could influence a more bitter taste, which is consistent with the findings of Kojic [71] in the development of snacks enriched with betalains. This bitterness can also be attributed to the presence of saponins and phenolic compounds in plant-based foods, which are responsible for attributes such as acidity, astringency, and bitterness [72].

## 4. Conclusions

The results of this study underscore the potential of beetroot stems and leaves as valuable nutritional resources. By optimizing the drying process, we can preserve key bioactive compounds, making these often-discarded parts suitable for fortifying various food products. The significant protein, dietary fiber, and mineral content, alongside their substantial amino acid contributions, position beetroot stems and leaves as excellent candidates for enhancing human nutrition. Furthermore, their rich betalain and polyphenol profiles add functional benefits. The incorporation of these components into food matrices not only addresses food waste reduction but also promotes health and nutrition, offering a sustainable and economical solution. Further investigations should explore the sensory qualities and consumer acceptance of different food products containing beetroot stem and leaf flour, as well as their long-term health impacts. Additionally, studying the scalability of incorporating these by-products into various food industries could enhance their practical application and contribute to broader sustainability goals.

## Figures and Tables

**Table 1:** Tortilla formulation.

Formulation	Cornmeal (%)	Beetroot Stem Flour (%)	Beetroot Leaf Flour (%)
Snack 1	100	-	-
Snack 2	90	5	5
Snack 3	80	10	10

**Table 2:** Effect of drying temperature on water activity and proximate composition.

Component (g/100 g) d.m	Leaves									Stems								
	50 °C			60 °C			70 °C			50 °C			60 °C			70 °C		
Water Activity	0.5036	±	0.0013 ^c^	0.4278	±	0.0008 ^b^	0.2011	±	0.0148 ^a^	0.4957	±	0.0021 ^c^	0.2851	±	0.0011 ^b^	0.1963	±	0.0066 ^a^
Protein	30.39	±	0.23 ^c^	30.54	±	0.98 ^c^	30.24	±	0.86 ^c^	15.24 ^b^	±	0.39	14.20	±	0.01 ^a^	14.44	±	0.51 ^a^
Lipids	4.0	±	0.02 ^c^	4.1	±	0.03 ^b^	3.5	±	0.02 ^a^	0.46	±	0.01 ^b^	0.53	±	0.02 ^a^	0.44	±	0.02 ^b^
Phytic Acid	0.146	±	0.001 ^c^	0.162	±	0.003 ^b^	0.178	±	0.004 ^a^	0.133	±	0.006 ^d^	0.118	±	0.001 ^e^	0.077	±	0.001 ^f^
Total Dietary Fiber	30.74	±	1.68 ^a^	27.09	±	6.73 ^ab^	30.42	±	1.07 ^ab^	32.88	±	2.24 ^b^	33.51	±	0.424 ^b^	32.71	±	2.04 ^b^
Ash	23.72 ^a^	±	0.12	24.15 ^a^	±	0.02	24.21 ^ab^	±	0.04	24.88 ^c^	±	0.01	25.43 ^bc^	±	0.18	25.47 ^bc^	±	0.07
E.N.N	12.45			15.36			12.75			25.53			25.36			26.10		

The values are expressed as mean ± standard deviation of triplicate measurements (*n* = 3). The data are expressed in dry basis (d.b). Different lowercase letters indicate significant differences according to Tukey’s multiple range test (*p* < 0.05). E.N.N: Calculated on the basis of 100-moisture-protein-lipid-ash and later corrected by humidity to declare the dry weight.

**Table 3:** Amino acid profile in stems and leaves of beetroot dried at different temperatures.

	Leaves (50 °C)			Leaves (60 °C)			Leaves (70 °C)			Stems (50 °C)			Stems (60 °C)			Stems (70 °C)		
Essential aminoacids																		
Histidine	0.216	±	0.009 ^b^	0.125	±	0.008 ^a^	0.203	±	0.002 ^b^	0.083	±	0.005 ^a^	0.082	±	0.002 ^a^	0.099	±	0.002 ^a^
Threonine	0.466	±	0.015 ^b^	0.464	±	0.008 ^b^	0.418	±	0.000 ^a^	0.144	±	0.003 ^b^	0.138	±	0.005 ^ab^	0.123	±	0.002 ^a^
Valine	0.065	±	0.002 ^a^	0.051	±	0.006 ^a^	0.047	±	0.001 ^a^	0.000	±	0.000	0.000	±	0.000	0.000	±	0.000
Methionine	0.009	±	0.003 ^a^	0.022	±	0.001 ^c^	0.018	±	0.001 ^b^	0.003	±	0.000 ^b^	0.002	±	0.000 ^a^	0.002	±	0.000 ^a^
Cysteine	0.049	±	0.000 ^a^	0.060	±	0.004 ^b^	0.039	±	0.000 ^c^	0.039	±	0.003 ^a^	0.032	±	0.003 ^a^	0.065	±	0.001 ^b^
Isoleucine	0.475	±	0.002 ^c^	0.410	±	0.001 ^b^	0.365	±	0.000 ^a^	0.173	±	0.001 ^b^	0.161	±	0.002 ^b^	0.103	±	0.005 ^a^
Leucine	0.851	±	0.005 ^c^	0.670	±	0.004 ^b^	0.608	±	0.000 ^a^	0.287	±	0.002 ^b^	0.266	±	0.003 ^b^	0.151	±	0.007 ^a^
Phenylalanine	0.524	±	0.008 ^c^	0.486	±	0.003 ^b^	0.436	±	0.004 ^a^	0.168	±	0.007 ^b^	0.157	±	0.001 ^b^	0.102	±	0.002 ^a^
Tyrosyne	0.821	±	0.002 ^c^	0.713	±	0.006 ^b^	0.608	±	0.006 ^a^	0.286	±	0.001 ^b^	0.269	±	0.001 ^b^	0.169	±	0.011 ^a^
Lysine	0.631	±	0.003 ^c^	0.565	±	0.006 ^b^	0.521	±	0.004 ^a^	0.219	±	0.009 ^b^	0.202	±	0.001 ^b^	0.134	±	0.003 ^a^
EAAs	4.106			3.565			3.263			1.402			1.308			0.947		
Non-essential aminoacids																		
Alanine	0.744	±	0.002 ^c^	0.707	±	0.001 ^b^	0.633	±	0.006 ^a^	0.299	±	0.010 ^ab^	0.336	±	0.013 ^b^	0.259	±	0.010 ^a^
Arginine	0.489	±	0.001 ^a^	0.470	±	0.014 ^a^	0.439	±	0.002 ^a^	0.013	±	0.000 ^a^	0.018	±	0.000 ^b^	0.013	±	0.001 ^a^
Aspartic acid	1.228	±	0.028 ^c^	1.003	±	0.020 ^b^	0.878	±	0.005 ^a^	0.405	±	0.002 ^c^	0.388	±	0.002 ^b^	0.271	±	0.002 ^a^
Glutamic acid	1.668	±	0.020 ^c^	1.371	±	0.020 ^b^	1.188	±	0.004 ^a^	0.692	±	0.025 ^b^	0.550	±	0.013 ^a^	0.470	±	0.019 ^a^
Glycine	0.700	±	0.006 ^b^	0.744	±	0.013 ^b^	0.673	±	0.005 ^a^	0.297	±	0.004 ^b^	0.303	±	0.004 ^b^	0.278	±	0.001 ^a^
Hydroxiproline	0.071	±	0.002 ^b^	0.065	±	0.001 ^a^	0.069	±	0.001 ^ab^	0.181	±	0.001 ^b^	0.161	±	0.001 ^a^	0.154	±	0.004 ^a^
Serine	0.511	±	0.028 ^b^	0.508	±	0.010 ^b^	0.464	±	0.000 ^a^	0.228	±	0.003 ^c^	0.205	±	0.003 ^b^	0.186	±	0.003 ^a^

Values are expressed as mean ± standard deviation of triplicate measurements (*n* = 3). The data are expressed in dry basis (d.b). Different lowercase letters indicate significant differences according to Tukey’s multiple range test (*p* < 0.05); EAAs—essential amino acids.

**Table 4:** Fatty acids profile in stems and leaves of beetroot dried at different temperatures.

Parameter	Lipid Number	Units	Leaves 50 °C			Leaves 60 °C			Leaves 70 °C			Stems 50 °C			Stems 60 °C			Stems 70 °C		
Lipids		g/100 g	4			4.1			3.5			0.46			0.53			*0.44*		
Total saturated fatty acids		g/100 g	0.8 ^a^			0.93 ^c^			0.88 ^b^			0.10 ^a^			0.10 ^a^			0.10 ^a^		
Total Polyunsaturated fatty acids		g/100 g	2.9 ^c^			2.84 ^b^			2.37 ^a^			0.32 ^b^			0.39 ^c^			0.30 ^a^		
Total monounsaturated fatty acids		g/100 g	0.30 ^b^			0.33 ^c^			0.25 ^a^			0.04 ^a^			0.04 ^a^			0.04 ^a^		
Fatty acid																				
Myristic	C14:0	g/100 g	0.04	±	0.00 ^a^	0.03	±	0.00 ^b^	0.03	±	0.00 ^b^	Traces			Traces			Traces		
Palmitic	C16:0	g/100 g	0.58	±	0.01 ^a^	0.69	±	0.00 ^c^	0.66	±	0.00 ^b^	0.08	±	0.00 ^a^	0.08	±	0.00 ^a^	0.08	±	0.00 ^a^
Margarico	C17:0	g/100 g	0.01	±	0.00 ^a^	0.01	±	0.00 ^a^	0.01	±	0.00 ^a^	Traces			Traces			Traces		
Stearic	C18:0	g/100 g	0.09	±	0.01 ^a^	0.11	±	0.01 ^c^	0.10	±	0.01 ^b^	0.01	±	0.00 ^a^	Traces			0.01	±	0.00 ^a^
Arachidic	C20:0	g/100 g	0.02	±	0.00 ^a^	0.03	±	0.00 ^b^	0.02	±	0.00 ^a^	Traces			Traces			Traces		
Behenic	C22:0	g/100 g	0.03	±	0.00 ^b^	0.03	±	0.00 ^b^	0.01	±	0.00 ^a^	Traces			Traces			Traces		
Lignocerico	C24:0	g/100 g	0.03	±	0.00 ^a^	0.03	±	0.00 ^a^	0.03	±	0.00 ^a^	0.01	±	0.00 ^a^	0.01	±	0.00 ^a^	Traces		
Other Unidentified	-	g/100 g	0.35	±	0.01 ^b^	0.49	±	0.03 ^c^	0.25	±	0.03 ^a^	0.02	±	0.00 ^a^	0.03	±	0.00 ^b^	0.02	±	0.00 ^a^
**Ʃ SFA**			0.8 ^a^			0.93 ^c^			0.88 ^b^			0.10 ^a^			0.10 ^a^			0.10 ^a^		
Palmitoleic acid	C16:1n7	g/100 g	Traces			0.01	±	0.00 ^a^	0.01	±	0.00 ^a^	Traces			Traces			Traces		
Oleic	C18:1n9	g/100 g	0.29	±	0.00 ^b^	0.31	±	0.00 ^c^	0.24	±	0.00 ^a^	0.04	±	0.00 ^b^	0.04	±	0.00 ^b^	0.03	±	0.00 ^a^
Eicosanoic acid	C20:1n9	g/100 g	0.01	±	0.00 ^a^	0.01	±	0.00 ^a^	0.01	±	0.00 ^a^	Traces			Traces			Traces		
**Ʃ MUFA**			2.9 ^c^			2.84 ^b^			2.37 ^a^			0.32 ^b^			0.39 ^c^			0.30 ^a^		
Linoleic acid (LA)	C18:2n6c	g/100 g	0.54	±	0.00 ^b^	0.57	±	0.00 ^c^	0.51	±	0.00 ^a^	0.19	±	0.00 ^a^	0.24	±	0.00 ^b^	0.19	±	0.00 ^a^
α-Linolenic acid (ALA)	C18:3n3	g/100 g	2.36	±	0.03 ^c^	2.26	±	0.03 ^b^	1.86	±	0.03 ^a^	0.13	±	0.00 ^b^	0.15	±	0.00 ^c^	0.10	±	0.00 ^a^
**Ʃ PUFA**			0.30 ^b^			0.33 ^c^			0.25 ^a^			0.04 ^a^			0.04 ^a^			0.04 ^a^		
Total omega 3		g/100 g	2.36 ^c^			2.26 ^b^			1.86 ^a^			0.13 ^b^			0.15 ^c^			0.10 ^a^		
Total omega 6		g/100 g	0.54 ^b^			0.57 ^c^			0.51 ^a^			0.19 ^a^			0.24 ^b^			0.19 ^a^		
LA/ALA	C18:2n6c/C18:3n3	g/g	0.22			0.25			0.27			1.46			1.6			1.9		

Adequate intake (AI) contribution expressed in energy percentage (%E) for LA and ALA for adults (≥18 age). The values are expressed as mean ± SD of three replicated determinations. The data are expressed in dry basis (d.b). Different lowercase letters indicate significant differences according to Tukey’s multiple range test (*p* < 0.05). SFAs—saturated fatty acids. MUFAs—monounsaturated fatty acids. PUFAs—polyunsaturated fatty acids.

**Table 6:** Functional properties of beetroot by-products.

Sample		WA_d_C * (g Water/g Sample)			WA_b_C * (g Water/g Sample)			WHC * (g Water/g Sample)			OHC * (g Oil/g Sample)		Solubility (%)												
														30 °C			60 °C			70 °C			80 °C		
Leaves	50 °C	0.631	±	0.036 ^a^	5.93	±	0.75 ^bc^	5.17	±	0.10 ^a^	2.29	±	0.06 ^c^	41.63	±	1.19 ^b^	33.10	±	1.62 ^c^	39.53	±	0.74 ^a^	40.70	±	2.62 ^ab^
	60 °C	0.631	±	0.002 ^a^	5.40	±	0.17 ^ab^	5.17	±	0.12 ^a^	2.17	±	0.09 ^ab^	40.69	±	1.44 ^ab^	36.00	±	1.26 ^c^	39.82	±	0.37 ^a^	38.88	±	1.88 ^a^
	70 °C	0.618	±	0.006 ^a^	5.22	±	0.73 ^a^	5.09	±	0.19 ^a^	2.19	±	0.03 ^bc^	37.75	±	1.61 ^a^	36.42	±	1.34 ^c^	38.31	±	1.28 ^a^	42.56	±	1.39 ^bc^
Stems	50 °C	1.28	±	0.02 ^b^	7.27	±	0.25 ^d^	8.15	±	0.31 ^b^	2.55	±	0.11 ^d^	45.14	±	1.74 ^c^	47.84	±	1.58 ^a^	48.98	±	1.07 ^b^	44.78	±	1.72 ^cd^
	60 °C	1.32	±	0.01 ^b^	6.36	±	0.23 ^c^	7.66	±	0.30 ^c^	2.21	±	0.01 ^bc^	47.45	±	1.85 ^c^	48.19	±	0.46 ^b^	48.90	±	1.79 ^b^	48.35	±	1.78 ^e^
	70 °C	1.370	±	0.04 ^c^	6.06	±	0.14 ^c^	6.01	±	0.20 ^d^	2.09	±	0.04 ^a^	46.88	±	2.31 ^c^	48.96	±	0.87 ^b^	46.31	±	0.88 ^b^	46.15	±	0.86 ^de^

(*) WA_d_C—water adsorption capacity; WA_b_C—water absorption capacity; WHC/OHC—water/oil holding capacity. Different letters indicate significant differences (*p*< 0.05) according to the Tukey’s test.

**Table 7:** Color parameters.

CieLab	Leaves									Stems								
	50 °C			60 °C			70 °C			50 °C			60 °C			70 °C		
L*	54.68	±	0.31 ^c^	52.13	±	0.50 ^b^	50.17	±	1.47 ^a^	49.45	±	0.31 ^a^	50.19	±	0.60 ^a^	52.11	±	0.33 ^b^
a*	−8.79	±	0.14 ^a^	−8.53	±	0.38 ^ab^	−8.14	±	0.27 ^b^	15.52	±	0.47 ^e^	12.61	±	0.20 ^d^	6.72	±	0.45 ^c^
b*	15.57	±	0.08 ^d^	15.33	±	0.72 ^d^	15.53	±	1.19 ^d^	0.86	±	0.08 ^a^	3.22	±	0.18 ^b^	6.37	±	0.18 ^c^

L*: luminosity; a*: red to green; b*: yellow to blue. Mean SD (*n* = 3). Different lowercase letters indicate significant differences according to Tukey’s multiple range test (*p* < 0.05).

**Table 8:** Effects of drying temperature on total phenolic content, antioxidant capacity, and nitrate content.

Parameters	Leaves												Stems					
	50 °C			60 °C			70 °C			50 °C			60 °C			70 °C		
TPC ^(1)^ (mg GAE/100 g)	558.9	±	5.3 ^b^	512.3	±	27.4 ^a^	478.2	±	12.9 ^a^	365.3	±	25.1 ^a^	362.2	±	69.8 ^a^	294.7	±	6.2 ^a^
DPPH ^(2)^ (µmol TE/100 g)	2057.24	±	1023.8 ^a^	3860.53	±	480.5 ^b^	3811.92	±	198.7 ^b^	2561.96	±	529.5 ^a^	3949.78	±	552.2 ^a^	2839.92	±	1108.7 ^a^
Nitrate (mg/kg)	159	±	0 ^c^	126	±	0 ^b^	100	±	0 ^a^	359	±	0 ^c^	314	±	0 ^b^	126.5	±	0.5 ^a^

(1) Galic acid equivalents (GAEs); (2) Trolox equivalent (TE). The values are expressed as mean ± standard deviation of triplicate measurements (*n* = 3). Different lowercase letters indicate significant differences according to Tukey’s multiple range test (*p* < 0.05).

**Table 9:** Sensory attributes of snacks baked with beetroot by-products.

Parameters	Control			10%			20%		
Color	4.5	±	0.7 ^c^	3.8	±	1.1 ^b^	3	±	0.9 ^a^
Flavor	3.8	±	0.9 ^ab^	4	±	1.0 ^c^	3.3	±	1.0 ^a^
Texture	3.6	±	0.8 ^a^	3.6	±	1.2 ^a^	3.3	±	1.0 ^a^
General acceptability	3.8	±	0.8 ^b^	3.8	±	1.1 ^b^	3.1	±	1.0 ^a^

Different proportions of beetroot waste flour were evaluated compared to the control sample with only corn flour. Different lowercase letters indicate significant differences according to Tukey’s multiple range test (*p* < 0.05).

## Data Availability

The original contributions presented in the study are included in the article, further inquiries can be directed to the corresponding author.
